# Supplementary material for: Chemosensory Receptors in Vertebrates: Structure and Computational Modeling Insights
Source: Int J Mol Sci. 2025 Jul 10;26(14):6605. doi: 10.3390/ijms26146605 (PMC12294341; doi:10.3390/ijms26146605)
Supplement: Supplementary file 1 [file ijms-26-06605-s001.zip › Supplementary Materials Figure S1.pdf]

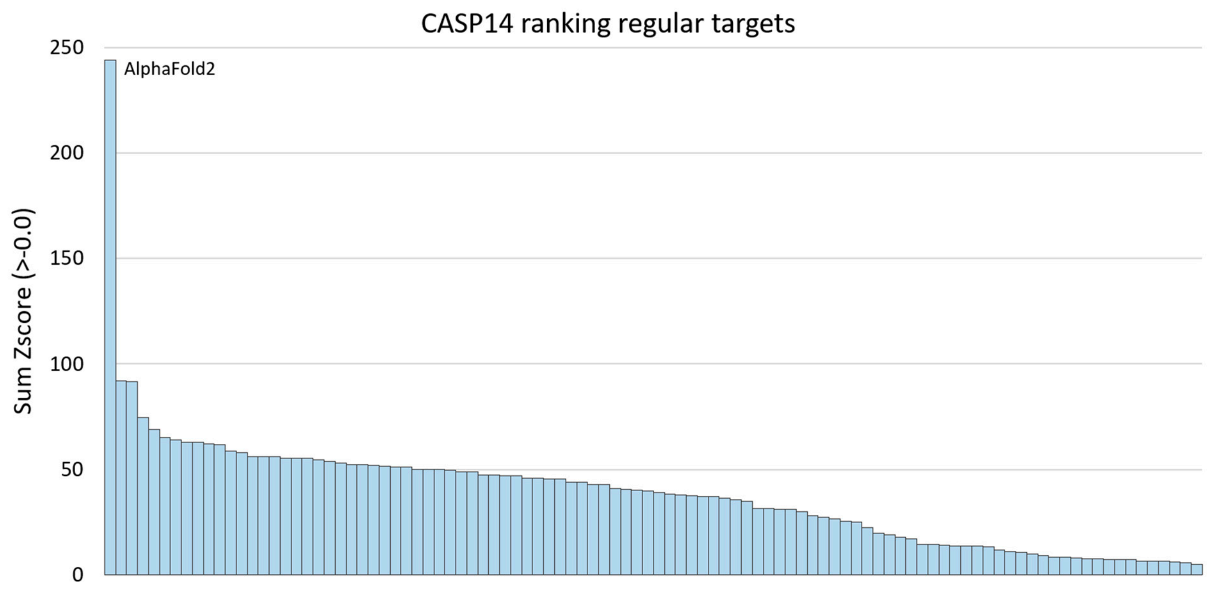

Figure S1: Performance of the top 100 groups in regular targets in the CASP14 experiment based on the sum of positive Zscores.

Data were collected on https://predictioncenter.org/casp14/zscores_final.cgi et visualized with excel.
