# Supplementary material for: Chemosensory Receptors in Vertebrates: Structure and Computational Modeling Insights
Source: Int J Mol Sci. 2025 Jul 10;26(14):6605. doi: 10.3390/ijms26146605 (PMC12294341; doi:10.3390/ijms26146605)
Supplement: Supplementary file 1 [file ijms-26-06605-s001.zip › Supplementary Materials Information File S1.pdf]

4. In silico study of structures

4.1. Modeling theories

4.1.2. Fold recognition or threading

- Profile–profile alignment: distant homologs can be found by comparing the profile of the target with the profiles of different families. The main difference between the different profile–profile methods lies in the algorithm used to compare the profiles. The methodology can be summarized into four steps: multiple sequence alignment, profile prediction, profile–profile alignment, and estimation of the score of these alignments.
- Structural profile: one of the pioneering methods in the inverse protein folding method was the use of the 3D-1D score to search for sequences that can fold like a known structure. The additional information can be added using two methods: the frozen approximation, with the score based on the template, and the defrosted approximation, with the score based on the template updated with the target sequence that affects the environment of the residues.

4.1.3. Ab initio or template-free

Folding the protein in steps: various algorithms can be used to fold the protein to obtain the minimum of energy for example, the Monte Carlo method or conformational space annealing (CSA). Sampling methods such as replica-exchange molecular dynamics (REMD) can be used to explore more configurations in a given simulation time. In these different methods, the protein can be simplified in a representation called coarse-grained to reduce the complexity of the computation. In the first simplification model, each amino acid is represented by two centers, representing Cα and the centroid of the side chain. The reduction in computation time allows for the possibility of not adding any knowledge-based information about the structure, which is required for a general structure prediction tool.

Foldit: the game involves moving the protein by adding constraints, changing the strength of freedom, and performing different energy minimizations and automatic moves to obtain the structure with the lowest energy. The involvement of humans allows a larger conformational space to be explored by overcoming energy barriers when needed, where algorithms can become stuck in a local minimum of energy. It also permits the exploration of methods unexplored by scientists before.

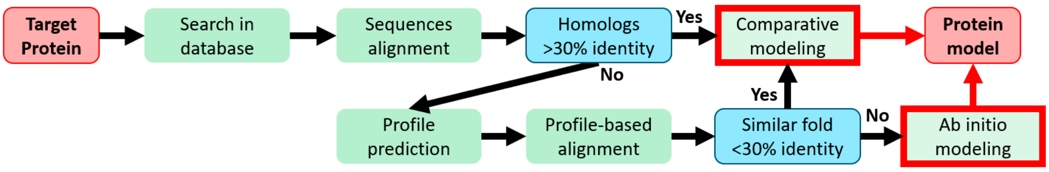

Figure 1. Decision diagram for structure modeling.

Researchers must decide which type of algorithm is best for their targets. This decision depends very much on the identity with structures already obtained experimentally (Figure 1). Indeed, the quality of the results is better with structure-based methods, so ab initio methods should only be used if no structure or fold with a good identity can be found.

4.1.4. Deep learning methods

In end-to-end methods every step is dedicated and optimized to the overall objective, and human intervention is reduced to obtain a purely data-driven algorithm. The first algorithms coupled distance-based prediction, attention, and end-to-end methods starting from the sequence and evolution

information encoded in the MSA. Then, the development of single-sequence-based algorithms improved and simplified the architecture to extract evolutionary information from the sequence only, instead of using MSA, with less computational time. This can be achieved by improving protein language models and using attention-based transformers to learn evolutionary information from the database. These new methods allow efficient models to be created without similar protein sequences by combining a pre-trained self-supervised protein language model and a geometry-inspired transformer model, which is faster than the previous state-of-the-art algorithm for the same-quality outputs.

4.1.5. Algorithm comparison

4.2. Model assessments

CASP: Several categories are evaluated: assembly modeling (complex), template-based modeling, ab initio modeling, contact prediction, refinement, and data-assisted modeling

CAMEO: It complements CASP, which features several categories, too: protein structure prediction, 3D protein structure quality assessment, and the prediction of macromolecular complexes (beta version)

4.3. The main modeling algorithms

4.3.1. Swiss-Model

Over the past 30 years, the algorithm has been continuously improved. The last functionality added was the ability to model homo- and heteromeric complex. The sequence input is accepted in many formats (sequence, FASTA, text, etc.).

4.3.2. Modeller

The constraints involved in modeling are the following:

- Bond length, bond angle, and dihedral angle;
- Van der Waals interactions;
- Disulfide bonds;
- $C\alpha$-$C\alpha$ distances and N-O distances in the backbone;
- Main-chain conformation, corresponding to six parts of the Ramachandran plot;
- Side-chain conformation (residue, type, h-bond, structure II, solvent accessibility);
- Prediction of dihedral angle conformation class.

4.3.3. Rosetta

Currently, web servers are dedicated to protein structure prediction with Rosetta algorithms. The different methods developed by the group can be used: RoseTTAFold (deep learning), RosettaCM (comparative modeling) [258], RosettaAB (ab initio), and prediction domains on the server developed by the Baker lab. We note that it is recommended to use RoseTTAFold, which outperforms the other methods. TrRosetta is another deep learning method using Rosetta, available on another server developed by the Yang lab; it was ranked first in regular targets in CASP 15 and protein domains in CASP 16 [253]

4.3.5. TASSER

I-TASSER followed the same steps with the addition of a second round of Monte Carlo simulation on the centroids of the clusters obtained after the first round. Some improvements were also introduced, including the identification of fragments by profile–profile alignment and the addition of a neural network to predict the accessible surface area.

4.3.8. AlphaFold

Another metric used is the SCASP14 score, which is the weighted sum of all the metrics used by CASP14; the median SCASP14 of AlphaFold2 is 2,2, compared to 1,01 for BAKER.

AlphaFold3 has the advantage of being able to produce high-quality results for all types of complexes, unlike many algorithms that are specialized in one task.

4.3.11. OmegaFold

OmegaFold is the first single-sequence-only-based algorithm that performs with the same quality as AlphaFold2 [237]. It has advantages in both time and independence for homologous sequences. Indeed, when the output quality decreases for the MSA-based methods without evolutionary information, OmegaFold maintains good accuracy. The model works with the same key concepts as language processing and deep neural networks. The difference is that the algorithm learns single- and pairwise-residue embeddings on unaligned sequences in a deep transformer-based protein language model, allowing it to obtain structural information. The second transformer used in OmegaFold is Geoformer, a geometry-inspired transformer neural network.

References

237. Wu, R.; Ding, F.; Wang, R.; Shen, R.; Zhang, X.; Luo, S.; Su, C.; Wu, Z.; Xie, Q.; Berger, B.; et al. High-Resolution *de Novo* Structure Prediction from Primary Sequence. *bioRxiv* 2022.

253. Du, Z.; Su, H.; Wang, W.; Ye, L.; Wei, H.; Peng, Z.; Anishchenko, I.; Baker, D.; Yang, J. The TrRosetta Server for Fast and Accurate Protein Structure Prediction. *Nature Protocols 2021 16:12* **2021**, *16*, 5634–5651, doi:10.1038/s41596-021-00628-9.

258. Rohl, C.A.; Strauss, C.E.M.; Chivian, D.; Baker, D. Modeling Structurally Variable Regions in Homologous Proteins With Rosetta. **2004**, doi:10.1002/prot.10629.
